# 21 days of mammalian omega-3 fatty acid supplementation improves aspects of neuromuscular function and performance in male athletes compared to olive oil placebo

**DOI:** 10.1186/s12970-015-0089-4

**Published:** 2015-06-18

**Authors:** Evan J. H. Lewis, Peter W. Radonic, Thomas M. S. Wolever, Greg D. Wells

**Affiliations:** Department of Nutritional Sciences, Faculty of Medicine, University of Toronto, 150 College St., M5S 3E2 Toronto, Ontario Canada; Faculty of Kinesiology & Physical Education, University of Toronto, 100 Devonshire Place, M5S 2C9 Toronto, Ontario Canada; Department of Physiology and Experimental Medicine, The Hospital for Sick Children, 555 University Ave, M5G 1X8 Toronto, Ontario Canada

**Keywords:** Omega-3 fatty acids, Neuromuscular function, Fat supplementation, Performance, Adaptations to training

## Abstract

**Background:**

Omega-3 polyunsaturated fatty acids (N-3) are essential nutrients for human health and integral components of neural tissues. There is evidence that N-3 supplementation may benefit exercise performance, however, no study has investigated the ergogenic potential of N-3 supplementation. Our objective was to determine the effect of short-term N-3 supplementation on neuromuscular-function and physical-performance in well-trained athletes.

**Methods:**

Male athletes (*n* = 30), 25 years (SD 4.6), training 17 h^.^wk^−1^ (SD 5) completed this randomized, placebo-controlled, parallel-design study. At baseline a blood sample was collected, maximal voluntary isometric contractions (MVC) with electromyography (EMG) recordings were measured, and participants underwent various performance tests including a Wingate test and 250 kJ time trial (TT) followed by repeated MVC and EMG measurement. Participants were then randomly assigned to receive N-3 (5 ml seal oil, 375 mg EPA, 230 mg DPA, 510 mg DHA) or placebo (5 ml olive oil) for 21-days after which baseline testing was repeated. The magnitude-based inference approach was used to estimate the probability that N-3 had a beneficial effect on neuromuscular-function and performance of at least ±1 %. Data are shown as mean ± 90 % confidence-interval.

**Results:**

Plasma EPA was higher on N-3 than placebo (*p* = 0.004) but the increases in DPA and DHA were not significant (*p* = 0.087, *p* = 0.058). N-3 supplementation had an unclear effect on MVC force (4.1 ± 6.6 %) but increased vastus lateralis EMG by 20 ± 18 % vs placebo (very likely beneficial). N-3 supplementation reduced Wingate percent power drop by 4.76 ± 3.4 % vs placebo (very likely beneficial), but the difference in TT performance was unclear (−1.9 ± 4.8 %).

**Conclusion:**

Our data indicates N-3 PUFA supplementation improved peripheral neuromuscular function and aspects of fatigue with an unclear effect on central neuromuscular function. Clinical trial registration NCT0201433.

## Background

The ability of skeletal muscles to generate force and resist fatigue is essential to sport performance. Training adaptations within the neuromuscular and skeletal muscle systems modulate muscle force generating capacity and the resistance to fatigue. Much investigation has occurred to determine the effect of nutritional supplements on the adaptations of skeletal muscle to strength training (e.g., protein supplementation) and methods to support endurance performance (e.g., carbohydrate loading). To date, however, there has been limited investigation on the effect of nutritional supplements on the neuromuscular system.

Central and peripheral nerves are comprised of fatty acids which are predominantly polyunsaturated [1]. Omega-3 (N-3) polyunsaturated fatty acids (PUFAs) are an integral component of neurons, nerve endings, myelin and muscle membranes [1]. N-3 PUFAs are essential nutrients which must be provided by the diet due to the inability of the body to synthesize them endogenously. The shortest N-3 PUFA is alpha linoleic acid (ALA; 18:3) found in seeds and nuts. Eicosapentaenoic acid (EPA; 20:5) and docosahexaenoic acid (DHA; 22–6) are longer chain N-3 PUFAs found in marine sources (e.g., fatty fish, seal). Studies on the effects of N-3 PUFA supplementation have predominantly focused on their potential ability to reduce cardiovascular risk factors [2]; however, there is growing evidence that N-3 PUFAs might support neural function [3, 4] and adaptations to exercise [5, 6]. Deficiencies in dietary N-3 PUFAs lead to reduced Na^+^/K^+^ ATPase activity and higher stimulation intensity for signal conduction [1]. In contrast, N-3 PUFA supplementation in different clinical and applied settings has enhanced nerve conduction velocity [3], membrane fluidity, sensitivity to acetylcholine [7] and also reduced post-exercise inflammation [5, 8].

The effect of N-3 PUFA supplementation on neuromuscular and physical adaptations to resistance training was studied in postmenopausal sedentary women who were randomly assigned to one of 3 groups: N-3 PUFA supplementation for 60-days followed by 90-days resistance training with N-3 PUFA supplementation (2 g^.^d^−1^ of fish oil), resistance training with N-3 PUFA supplementation or resistance training with no supplementation [6]. Supplementation without training over 60-days had no effect neuromuscular function or physical tests. After training all groups showed improvements in neuromuscular function and physical test; however, the N-3 PUFA groups showed significantly greater quadriceps peak toque and muscle EMG activation and the time delay between increased muscle electrical activity and contraction onset was decreased. These findings provide evidence of the ergogenic potential of this nutritional supplement. Since Lui et al. [9] found that N-3 PUFA were incorporated into muscle membrane after 21-days of supplementation, we hypothesized that 21-days of N-3 PUFA supplementation would have an effect on neuromuscular function and physical performance.

Therefore, our objective was to determine if short-term N-3 PUFA supplementation has an ergogenic effect through adaptations in the neuromuscular system. The primary endpoint of this study was change in quadriceps maximal voluntary contraction force. The secondary endpoints were change in muscle activation during quadriceps maximal voluntary contractions and a series of performance tests.

## Methods

**Fig. 1:**
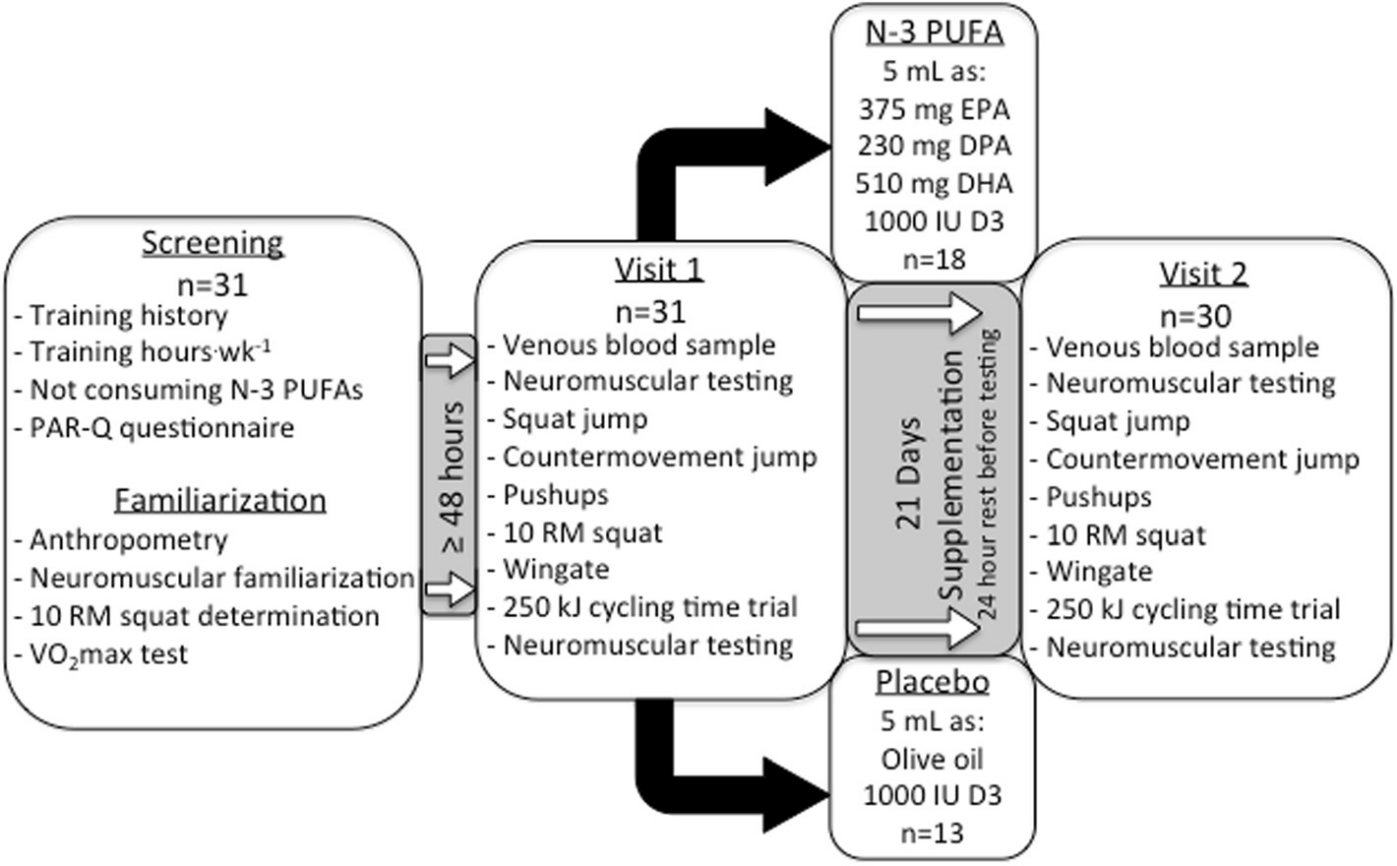
Experimental design

Thirty-one healthy male athletes were recruited for this parallel design, placebo controlled study. A study overview is shown in Fig. 1. Participant’s descriptive characteristics are given in Table 1. All competed in summer Olympic sports (e.g., rowing, sailing, triathlon, running) that require well developed strength-endurance and endurance. Participants were screened for eligibility based on sport specific competitive history (>2 years), training hours per week (>12 h), performing back squats as part of their regular training and not consuming any form of N-3 PUFA supplement or consuming fish ≥ 3 times per week for 4-weeks prior to beginning the study. The PAR-Q exercise readiness questionnaire was used for medical screening (www.csep.ca/english/view.asp?x=698). All participants were informed of study procedure and provided written consent. Approval for this project was obtained from the University of Toronto Research Ethics Board.

**Table 1:** Descriptive data for study participants in the omega-3 supplementation group (N-3 PUFA) and placebo group. Independent *t*-test determined no difference between groups

Measure	N-3 PUFA	Placebo	*p*
	(*n* = 18)	(*n* = 12)	
Height (cm)	180.3 ± 7.5	180.0 ± 7.7	0.91
Weight (kg)	79.8 ± 8.0	80.2 ± 10.8	0.69
Age (y)	23.7 ± 4.9	26.0 ± 3.0	0.21
Body Fat (%)	12.4 ± 3.3	12.9 ± 4.4	0.74
VO2max (ml ^.^ kg^-1 .^ min^−1^)	49.3 ± 14.2	48.9 ± 3.4	0.93
10RM Squat Max (kg)	92.7 ± 23.2	97.7 ± 16.8	0.40
Watt max	350 ± 47.4	345 ± 35.2	0.86
Training (h ^.^ wk^−1^)	17 ± 6	16 ± 5	0.64

### Pre-experimental procedures

Height, weight and body fat percentage (Omron Fat Loss Monitor, model HBF-306CAN, Omron Healthcare, Bannockburn, Illinois) were measured wearing lightweight shorts. After a 10-min cycling warm-up at 80 W, participants were fitted to a custom-made isometric dynamometer. Velcro straps were used to restrain the chest and hips. Quadriceps muscle force was measured from a load cell (SM-500-I9; Durham Instruments, Pickering, Canada) attached in series to the malleolus of the right ankle. The knee and trunk-thigh angle was fixed at 90°. Force measurements were recorded using a PowerLab 8/35 (ADInstruments, Colorado Springs, CO).

### Neuromuscular testing

#### Electrical stimulations

Stimulations of the femoral nerve were delivered from a high-voltage (400 V) constant current stimulator (Biopac, BSLSTMA, Santa Barbara, California), controlled by a custom designed program (LabChart 7, ADInstruments, Colorado Springs, CO, USA). A square wave, 1-ms stimulation was delivered from a cathode (10 mm diameter) (Kendall 100, Covidien, Saint-Laurant, Quebec, Canada) placed over the femoral nerve at the femoral triangle beneath the inguinal ligament. The anode (5 x 10 cm, DJO, Vista, CA, USA) was on the lower portion of the gluteal fold opposite to the cathode. Determination of optimal stimulation intensity (67.6 ± 14.4 mA; range 41.5–89.6) was achieved by increasing stimulation intensity until a further 5 mA increase did not alter peak quadriceps twitch force and vastus lateralis M-wave amplitude.

#### Maximal voluntary contractions

Maximal voluntary isometric contraction (MVC) was used to determine peak muscle force from the dominant quadriceps. During each neuromuscular testing session, three 5-s MVCs were performed, each separated by 1-min rest. Participants were strongly encouraged during each trial. The best MVC was used for measurement of peak muscle force. The rate of force development (RFD) was calculated for each MVC and averaged. This was performed using a custom analysis program (Matlab 6.0; Mathworks Inc.) and defined as the slope of the force time curve between 20 and 80 % of peak force [6].

#### Maximal quadriceps activation

A superimposed high-frequency doublet (100 Hz; 10 ms inter-stimulus interval) was given at 2.5-s into the MVC and at 5-s after the contraction with the muscle in a potentiated state. Stimulation intensity was 120 % of the measured optimal stimulation intensity to ensure supra-maximal stimulation during the MVCs. The ratio of the amplitude of the superimposed twitch during the MVC over the potentiated twitch was used to calculate voluntary activation as follows:

$$ \mathrm{V}\mathrm{A}\left(\%\right) = \left(1\ \hbox{--}\ \mathrm{superimposed}\ \mathrm{twitch}\ /\ \mathrm{potentiated}\ \mathrm{twitch}\right)\ \mathrm{x}\ 100 $$

#### Electromyographic recordings

Skin on the vastus lateralis (VL) and the patella was shaved free of hair, lightly abraded with sandpaper and cleaned with alcohol to ensure low impedance (Z <5 kΩ). VL EMG signal was recorded using Ag-AgCl electrodes (Kendall 100, Covidien, Saint-Laurant, Quebec, Canada; inter-electrode distance of 25 mm) place lengthwise over the mid-belly of the muscle with the reference electrode placed over the patella [10]. EMG measurements were recorded at 2 kHz, using PowerLab 8/35 (ADInstruments, Colorado Springs, CO, USA). EMG signal was amplified with a Dual BioAmp amplifier (ADInstruments, Colorado Springs, CO, USA; bandwidth frequency 10–500 Hz input impedance 200 MΩ, common mode rejection ratio = 85 dB, gain = ±1 %), transmitted to a PC and analyzed using a custom analysis program (Matlab 6.0; Mathworks Inc.).

Muscle activation was determined by calculating the EMG root mean square (RMS) from when muscle force began to plateau until immediately before the superimposed stimulation. RMS was averaged over 0.2-s periods. The mean of all three MVC was used for determining change. All RMS values were normalized to the pre-Visit 1.

The electromechanical delay (EMD) was calculated from the time difference from the increase in quadriceps EMG activity and force >10 standard deviations from resting baseline. This calculation was performed in our custom Matlab analysis program.

#### Determination of back squat 10 repetition maximum

After a series of submaximal warm-up sets, participants selected a weight thought to be their 10RM max based on current training weights. If the participant thought the weight was not maximal while lifting, they were encouraged to stop and additional weight was added using the methods of McLester et al. [11]. This process continued until a weight the participant could lift for 10 repetitions was determined. To standardize squat depth, an elastic band was tied across the back of the squat rack such that participants’ gluteal muscles would touch the band when the femur was parallel to the ground. Repetition only counted when contact was made with the band and band height was measured and kept constant for all visits.

#### Maximal oxygen uptake

Participants’ aerobic capacity was determined using breath-by-breath gas-analysis (MetaMax 3B, CORTEX, Leipzig, Germany) during an incremental cycling test on an ergometer (Lode Excalibur, Groningen, Netherlands). Resistance was set at 50 W, 100 W and 150 W for two minutes each before increasing by 25 W per minute thereafter. VO_2_max was determined as the highest valued achieved over a 20-s period. Watt max was determined from the stage at which participants’ cadence dropped below 50 rpm.

### Experimental protocol

The experimental protocol consisted of two identical testing sessions separated by 21-days of supplementation. Neuromuscular testing occurred before and after a series of performance tests to examine changes in neuromuscular function, performance and neuromuscular fatigue respectively.

#### Visit 1

Participants returned to the lab at least 48 h after completing the pre-experimental procedures. Participants were then weighed to determine Wingate test resistance, before warming-up on a cycling ergometer (Lode Excalibur, Groningen, Netherlands) for 10-min at 80 W.

Neuromuscular testing (pre-Visit 1) was then performed as described above. Next participants were allowed to stretch before performing 3 maximal squat jumps and countermovement jumps on a force plate (AccuPower ACP, AMTI, Watertown, MA, USA) to measure jump height using to the manufacturers software (AccuPower v1.6.3, AMTI, Waterdown, MA, USA).

After 5-min rest, participants completed as many pushups as possible in 1-min, were given 1-min rest and then repeated the test a second time. Participants entered a pushup position with elbows at 90° flexion and an elastic band was placed in contact with chest to denote minimum depth. 1-min rest was given before repeating the protocol. This assessment was interpreted as a measure of upper-body strength and strength endurance.

After a 5 min break participants began to warm-up for the back squats by performing 4-6reps at 20 %, 40 %, 60 %, 80 % of their 10RM max before performing the maximum number of repetitions at 100 % of the predetermined 10RM max squat weight.

Participants cycled against light resistance for 10 min before performing a 30 s Wingate test (Monark 894, Vansbro, Sweden) at 7.5 % of bodyweight [12]. Peak power, average power and power drop (%) were recorded.

Participants recovered for 20-min with low intensity cycling before completing, a 250 kJ cycling time trial (Lode Excalibur, Groningen, Netherlands). The bike was set in a pedaling-dependent with power varied by cadence [13]. Resistance was set using 75 % of maximum power reached in the VO_2_max test according to the methods of Jeukendrup et al. [13]. Participants received no verbal feedback during the time trial regarding the distance they had covered and were could only see work completed as an indication of distance travelled. A standing fan was set at the same speed to reduce thermal stress.

As soon as reasonably possible after the time trial, participants returned to the isometric dynamometer to repeat neuromuscular testing (post-Visit 1). At the completion of testing, participants were given their randomly assigned supplement in a sealed opaque envelope.

#### Visit 2

Participants were asked to refrain from training for 24 h prior, to control for athletic recovery. All procedures were identical to Visit 1. Participants were not informed of prior test performances to ensure that their performance was not artificially enhanced through mental self-competition.

#### Training and dietary controls

Participants were recruited during their regular training season to ensure constant levels of activity. For the duration of the study, participants were instructed to maintain normal level of training, with the exception of resting for 24-h prior to each testing session. Participants were instructed to refrain from consuming >3 servings of fish per week, in addition to any non-supplied omega-3 supplements. At the beginning of Visit 1, participants completed a 24-h diet record. This was scanned and sent back to each participant prior to Visit 2 to ensure the same foods were eaten prior to each test. All testing took place at the same time of day to avoid temporal variation.

#### Omega-3 supplementations

For 21-days, participants consumed 5 mL of seal oil N-3 PUFA (5000 mg N-3 PUFA, 375 mg EPA, 230 mg DPA, 510 mg DHA and with 1000 IU vitamin D3) (Auum Inc., Timmons, On) or 5 mL olive oil (Bertolli, Mississauga, Canada) with 1000 IU vitamin D added. Participants were instructed to take 2–2.5 mL servings orally twice daily, and to let the oil remain in the mouth for 1-min before swallowing to allow for sublingual absorption [14].

Seal oil was chosen as the source of experimental N-3 supplement for this study because mammalian (seal) triacylglycerol molecules have N-3 PUFA fats primarily in the sn-1 and sn-3 positions, as opposed to the sn-2 position of fish oil N-3 PUFA [15, 16]. Fats in the sn-3 position are preferentially cleaved by sublingual lipases, and the sn-1 fat is cleaved in the small intestine, while the sn-2 fatty acid is left for later esterification [14, 17]. These structural differences are thought to enable chylomicrons and chylomicron remnants containing mammalian N-3 PUFA to have a higher rate of clearance from the blood compared to fish oil intake [18].

#### Randomization and concealment

Participants were assigned a sequential study ID based on their enrollment. Treatment assignment (n-3 PUFA or placebo) was determined from a computer generated random number sequence. Supplement bottles were then sealed in opaque envelopes and labeled with participant ID by a researcher not involved in the study. Participants received their supplement at the end of visit 1. Study researchers were unblinded after plasma n-3 analysis was completed.

#### Blood sampling

A resting 8 mL blood sample from the antecubital vein was collected into K3-EDTA Vacutainer tubes (BD Vacutainer, Mississauga, Canada) at the beginning of Visit 1 and 2. Samples were centrifuged (3000 rpm for 15 min at 4 °C). Plasma samples were collected and stored at −80 °C for later analysis of plasma N-3 PUFA concentration.

#### Lipid extraction and gas chromatography-flame ionization detection

All lipid extraction techniques have been previously described by Chen et al. [19]. To extract total lipids, Folch, Lees, and Sloane Stanley’s method was applied by use of chloroform: methanol: 0.88 % KCl (2: 1: 0.75 by vol.) Total lipid extraction (TLE) with known quantity of heptadecanoic acid (17:0) was heated for 1 h at 100 °C with 14 % boron trifluoridemethanol to convert to fatty acid methyl esters (FAME; ester-linked fatty acids). Gas chromatography-flame ionization detection (GC-FID) was used to quantify FAME.

A Varian-430 gas chromatograph (Varian, Lake Forest, CA, USA) with a Varian FactorFour capillary column (VF-23 ms; 30 m · 0.25 mm i.d. · 0.25 lm film thickness) and an FID was used to analyze FAME injected in splitless mode. Injector and detector ports were set at 250 °C and helium carrier gas was set at a constant flow rate of 0.7 mL/min. A specific temperature program was used during FAME elution, it was set at 50 °C for 2 min, slowly increasing by 20 °C/min, and held at 170 °C for 1 min, then at 3 °C/min and held at 212 °C for 5 min to complete the run at 28 min. Retention times of authentic FAME standards (Nu-Chek Prep, Inc., Elysian, MN, USA) were used to identify peaks. Internal standard (17:0) peaks were compared to phospholipids peaks to calculate fatty acid concentrations, with final values expressed in nmol/g brain. Protocol for GC-FID was adopted from Chen et al. [19] where it is well described.

### Statistical analyses

This trial was registered at clinicaltrials.gov (NCT0201433) with statistical analysis of repeated measures ANOVA on all neuromuscular and performance measurements. After analysis had begun we became aware of the magnitude-based inferences analysis approach [20]. The authors determined this approach was more appropriate given our sample size and population of interest.

The standardized difference in means of neuromuscular measures was compared as follows: pre-Visit 1 to pre-Visit 2 and pre-Visit 2 to post-Visit 2. As well the standardized difference in means of performance tests from Visit-1 and Visit-2 were compared using pooled standard deviation as previously described by our lab [21]. The difference between time points was examined quantitatively using magnitude based inferences as described by Hopkins et al. [20].

A threshold of ±1.0 % was set as threshold for beneficial or harmful effects on performance as previously described [22]. The effect of supplementation was interpreted as unclear when the odds ratio (OR) of benefit ≥ harm was 66. This corresponds with an effect that is possibly beneficial (25 % chance of benefit) and most unlikely harmful (0.5 % risk of harm) [22].

The inference was generated from the confidence limits derived from the *p*-value associated with the effect and the magnitude of the difference in means such that a probability that the true value falls within the 90 % confidence interval corresponds with a qualitative inference of a 0 % “most unlikely” effect; 0.5 % “very unlikely”; 5 % “unlikely”; 25 % possibly; 75 % likely; 95 % likely; 99.5 % very likely; 100 % most likely. Where confidence intervals included values that were both positive and negative, OR are used to interpret if findings are meaningful and discussed further. Raw data is presented as mean ± SD for N-3 PUFA and PLA groups respectively. Interpreted data is presented as mean ± 90 % CI with corresponding qualitative inference and OR where appropriate.

Changes in blood N-3 PUFA concentration were analyzed using a repeated measures-ANOVA (Group x Time) and differences in descriptive statistics were analyzed using an unpaired *t*-test (SPSS v21, Armonk, NY). Significance was set at *p* <0.05.

## Results

One participant was unable to complete Visit 2 due to competition related travel. His data was removed from analysis and final analysis was performed with an *n* = 30.

When baseline characteristics were compared, the experimental N-3 PUFA and placebo groups were not different from each other.

### Neuromuscular measurements

#### MVC force

**Fig. 2:**
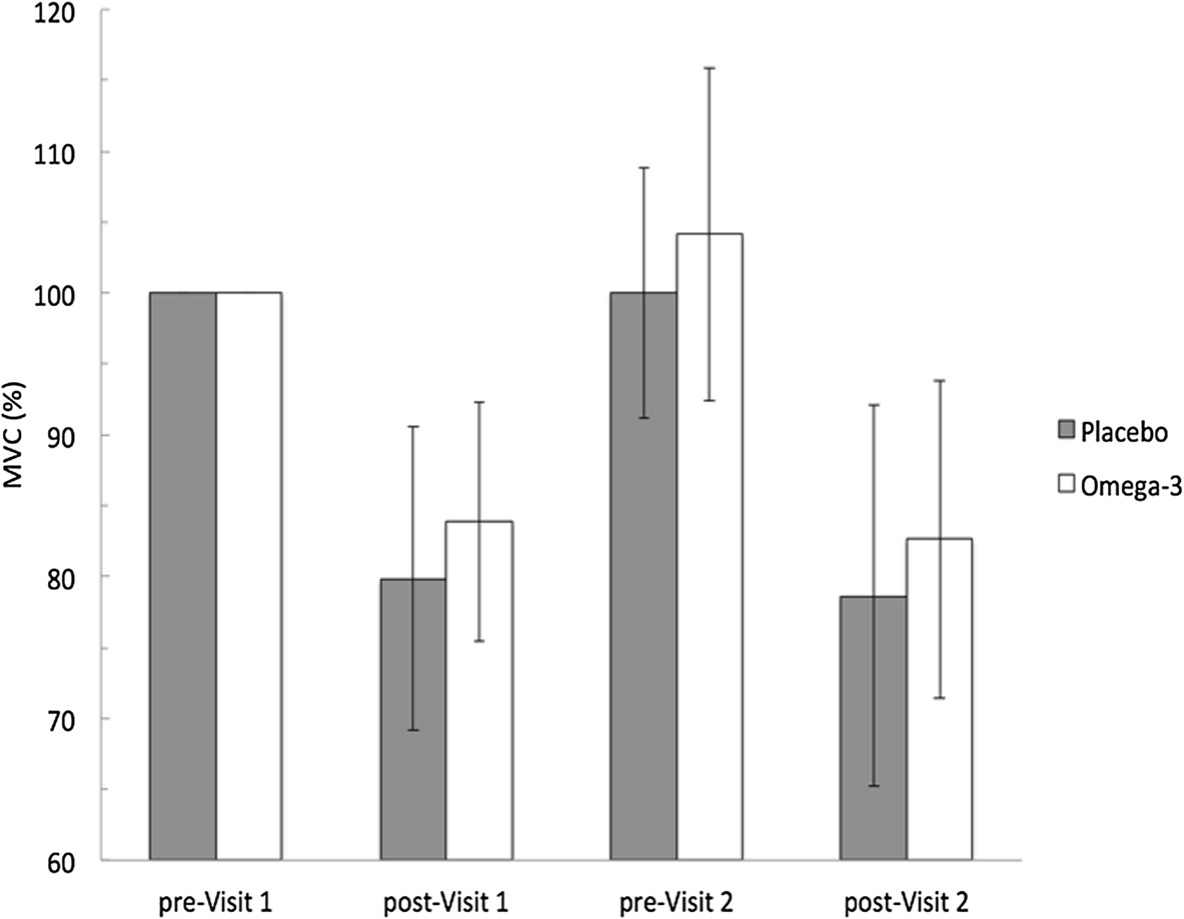
Change in quadriceps maximal voluntary contraction (MVC) force is shown as percent change from pre-visit 1. Data are shown as mean ± SD

On N-3 PUFA supplement quadriceps MVC force increased from 643 ± 144 to 670 ± 175 N (4.1 %), while on placebo force increased from 677 ± 107 to 683 ± 154 (0.03 %) (Fig. 2). This represents an unclear change of (4.1 % ± 6.6; OR = 30). When MVC force was compared from pre-Visit 2 to post-Visit 2, there was a similar decrease in force of −20.3 % and −21.2 % representing an unclear difference (0.95 % ± 7.5; OR = 2). Similarly, there was an unclear difference in force between post-Visit 1 and post-Visit 2 (0.90 % ± 8.6; OR = 2).

#### Rate of force development

Quadriceps RFD was measured during each MVC and averaged. On N-3 PUFA supplement MVC force increased from 2376 ± 709 N^.^s^−1^ to 2522 ± 929 (7.6 %), while on placebo 2101 ± 608 N^.^s^−1^ to 2354 ± 700 (17.1 %). This represents an unclear difference (9.5 % ± 22; OR = 11). Comparison of RFD from pre-Visit 2 to post-Visit 2 showed an similar change of −16.2 % ± 3.9 and −22.9 % ± 29.6 representing an unclear difference between groups (−6.7 % ± 14; OR = 15). When post-Visit 1 and post-Visit 2 were compared the N-3 PUFA group was −1.4 % ± 23.6 lower and PLA was 14.6 % ± 37.5 higher, representing a likely harmful effect (−16.0 % ± 19.0; OR = 0).

#### Electromyography and electromyography delay

**Fig. 3:**
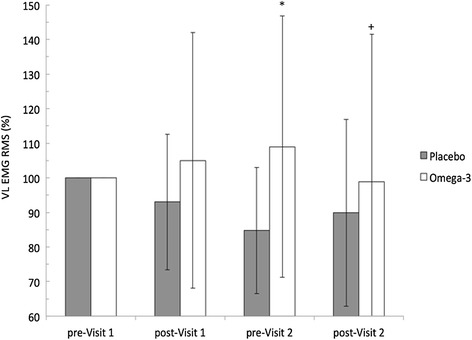
Change in vastus lateralis (VL) EMG RMS is shown as percent change from pre-Visit 1. *Very likely beneficial increase compared to placebo. + Very likely harmful effect compared to placebo. Data are shown as mean ± SD

Quadriceps muscle activation was calculated from the RMS EMG signal collected from the VL and normalized to pre-Visit 1 (Fig. 3). Participants on N-3 PUFA supplement increased muscle activation 9.0 % ± 37.8, while on placebo EMG decreased 12.9 % ± 19.4. This represents a very likely benefit (22.0 % ± 20.0; OR = 763). When RMS was compared from pre-Visit 2 to post-Visit 2, N-3 PUFAs had a likely harmful effect compared to placebo (−11.3 % ± 12; OR = 0), while the change between post-Visit 1 and post-Visit 2 represented an unclear difference (5.9 % ± 3.9; OR = 2).

On N-3 PUFA participants’ EMD increased from 0.053 ± 0.014 s to 0.055 ± 0.019 (16.5 %), while on placebo EMD was lower 0.050 ± 0.020 s to 0.042 ± 0.018 (−0.85 %). This represents an unclear difference (17.3 % ± 39.0; OR = 11). Comparison of EMD change between pre-Visit 2 to post-Visit 2 showed a likely beneficial effect of N-3 PUFA (−35.3 % ± 35.1; OR = 361), while there was an unclear difference between post-Visit 1 and post-Visit 2 (3.6 % ± 28.0; OR = 2).

#### Voluntary activation

Maximal quadriceps activation was tested using the interpolated twitch technique during each MVC and averaged. On N-3 PUFA, voluntary activation increased from 91.1 % ± 7.5 to 91.8 % ± 5. 6 (1.3 %), while on placebo increased from 89.5 % ± 8.9 to 92.2 % ± 3.7 (4.0 %). This represents an unclear change between time points (2.7 % ± 6.8). Comparison of voluntary activation from pre-Visit 2 to post-Visit 2 was unclear (0.26 % ± 4.7), as was post-Visit 1 to post-Visit 2 (0.050 % ± 3.4).

### Performance measures

Changes from Visit 1 to Visit 2 are shown in Table 2 to examine both raw differences and interpreted percent changes. Both groups have similar performances in Visit 1, showing homogeneity of the sample population.

**Table 2:** Performance measures Visit 1 and Visit 2 for the omega-3 supplementation (N-3 PUFA) and placebo groups. The differences in mean change is given as a percent ± 90 % confidence limits and corresponding inference

Test	N-3 PUFA		Placebo		ΔN-3 vs. Δ PLA (% ±90 % CL)	Inference
	Visit 1	Visit 2	Visit 1	Visit 2		
Squat Jump (cm)	34.6 ± 7.7	34.7 ± 6.7	36.6 ± 3.1	38.0 ± 7.1	2.4 ± 9.3	Unclear
Counter Movement Jump (cm)	43.5 ± 9.4	43.6 ± 7.2	45.5 ± 4.7	45.7 ± 6.2	1.4 ± 1.4	Unclear
Push-ups 1	50 ± 14	51 ± 13	49 ± 13	51 ± 14	1 ± 5.8	Unclear
Push-ups 2	23 ± 11	24 ± 10	25 ± 10	27 ± 12	1 ± 6.6	Unclear
Back Squat	13 ± 4	15 ± 4	12 ± 2	15 ± 4	5 ± 19	Unclear
Wingate PP (W)	841.30 ± 160.50	838 ± 176.84	835 ± 136.65	847.26 ± 130.25	2.17 ± 4.7	Unclear
Wingate Average Power (W)	593.1 ± 95.78	599.7 ± 92.65	611.3 ± 90.69	616.1 ± 104.44	0.74 ± 2.9	Unclear
Wingate Power Drop (%)	54.8 ± 9.54	54.6 ± 10.33	49.1 ± 5.82	53.0 ± 5.46	4.76 ± 3.4	Very likely beneficial
250 kJ Time Trial (sec)	1133.2 ± 296.1	1126.9 ± 265.7	1093.8 ± 269.8	1116.7 ± 269.6	1.96 ± 4.8	Unclear

Change in the number of repetitions of back squat was compared before and after supplementation. From pre-testing 10RM determination to Visit 1, there was an increase of 3 ± 4 and 2 ± 2 repetitions performed by the N-3 PUFA and placebo group respectfully.

A change in performance was measured in the Wingate test. The N-3 PUFA group Wingate power drop was lower from Visit 1 to Visit 2, while the PLA group showed an increase in fatigue. This was very likely beneficial (4.76 ± 3.4 %; OR = 6870).

In the 250 kJ TT the N-3 PUFA group mean was slower than the PLA group by 39.4 s. At Visit 2, the N-3 PUFA group decreased their time by 6.2 ± 131.6 s while the PLA group increased by 22.9 ± 65.1 s. With N-3 PUFA supplementation 9 participants improved time trial time by 105.7 ± 106.0 s, whereas 4 PLA participants improved by 39.2 ± 75.8 s.

### Plasma fatty acid analysis

Gas-flame chromatography performed to determine change in the percent composition of plasma fatty acids between groups (Table 3). Plasma EPA increased in the N-3 PUFA group with no change in PLA after supplementation (*p* = 0.004). There was a trend for higher DPA and DHA after supplementation (*p* = 0.087, *p* = 0.058).

**Table 3:** Fatty acid composition (%) of plasma for the omega-3 supplementation (N-3 PUFA) and placebo groups

	N-3 PUFA		Placebo		
Fatty acids	Pre	Post	Pre	Post	*p*
EPA (20:5 n-3)	0.41 ± 0.16	0.68 ± 0.27*	0.59 ± 0.34	0.49 ± 0.24	0.004
DPA (22:5 n-3)	0.29 ± 0.092	0.35 ± 0.091	0.31 ± 0.095	0.30 ± 0.055	0.087
DHA (22:6 n-3)	1.61 ± 0.32	1.81 ± 0.43	1.65 ± 0.45	1.56 ± 0.042	0.058
α-Linolenic (18:3 n-3)	0.39 ± 0.12	0.38 ± 0.18	0.41 ± 0.10	0.36 ± 0.11	0.53
Linoleic (18:2 n-6)	21.68 ± 2.85	21.01 ± 1.89	22.75 ± 3.67	21.93 ± 3.16	0.86
Arachidonic (20:4 n-6)	8.00 ± 2.07	7.43 ± 1.69	7.42 ± 1.93	7.03 ± 1.97	0.86

*Significant group by time interaction. All values are mean ± SD percentages for the omega-3 supplementation group (N-3 PUFA) and placebo group (PLA). Pre, data collected prior to 21-days of supplementation. Post, data collect after 21-day of supplementation. Data are shown as mean ± SD

## Discussion

This present study sought to elucidate the effect of 21-days of N-3 PUFA supplementation on neuromuscular function and performance measures in well-trained male athletes. To our knowledge this is the first study to evaluate the effects of a N-3 PUFA supplement on athletes while measuring changes in both neuromuscular function and performance. This study found that N-3 PUFA supplementation increased muscle activation and attenuated fatigue as assessed during a Wingate test after maximal back squat exercise by reducing percent power drop.

### Neuromuscular function

The primary outcome of this study was change in MVC force from pre-Visit −1 to pre-Visit 2. The N-3 PUFA group had an unclear 4.1 % ± 6.6 increase in MVC force, indicating that individuals can experience a beneficial or harmful change. However, we did observe a 9 % increase in VL EMG activation in the N-3 PUFA group compared to baseline and a very likely beneficial 22.0 ± 20.0 increase in muscle activation. These data suggest N-3 PUFA supplementation supported peripheral (neuromuscular junction to contractile apparatus) but not central (brain to neuromuscular junction) neuromuscular adaptations. In comparison, Rodacki et al. [6] showed that 2 g^.^d^−1^ fish oil N-3 PUFA supplementation for 90 days during resistance training in sedentary women increased muscle activation and MVC force by ~50 % over training only controls. Two differences between this work and the present study was supplement duration and dose of 90 days compared to 21 days and 2 g^.^d^−1^ compared to 1.1 g^.^d^−1^. Therefore, a longer supplementation period and/or a higher dose might be necessary for the observed increase in muscle activation to translate into a beneficial increase in muscle force in this highly trained population. These results along with Rodacki et al. [6] provide evidence of the effect N-3 PUFA supplementation on neuromuscular function across the training spectrum and suggest a beneficial relationship between N-3 PUFA supplementation and neuromuscular adaptations to training.

### Neuromuscular fatigue

When we examined the neuromuscular fatigue assessed by change from pre to post Visit 2, N-3 PUFA had a mixed effect. Central neuromuscular fatigue was apparent but not different between groups as MVC force was ~20 % lower in both groups and voluntary activation was unchanged. Peripheral measures showed no difference in quadriceps rate of force development, while EMD was lower, indicating a faster activation of the contractile apparatus. Interestingly, N-3 PUFA VL EMG activation in was 10 % lower compared to pre-Visit 2 testing suggesting peripheral neuromuscular fatigue. We suggest that the N-3 group performed more work during visit 2 as demonstrated by attenuated fatigue in the Wingate test and the unclear 1.96 % ± 4.8 difference in 250 kJ TT performance. Regardless, VL EMG at post-Visit 2 returned to baseline levels, therefore we interpret any potential harm with caution.

### Performance changes

Over the 21-day intervention, all participants were instructed to continue with their normal daily training. This was an important aspect of the study intervention, as results from Rodacki et al. [6] revealed N-3 PUFA supplementation only enhanced neuromuscular function during resistance training and not during normal activities of daily living. As expected, both groups showed evidence of training adaptations during the supplementation period. In the neuromuscular assessment, MVC RFD increased similarly between groups, while the number of back squat repetitions with 10RM weight was increased ~20 % from familiarization to Visit 1 and 2 respectively. The improvement in 10RM back squat repetitions caused 4.76 % ± 3.4 higher fatigue during the Wingate test in the placebo group. We did not observe any change in peak or mean Wingate power between visits, indicating no change in central neuromuscular function, however, the attenuation of fatigue observed in the N-3 group indicates maintained peripheral neuromuscular function. This finding is consistent with the observed increase in muscle activation. Performance in the 250 kJ time trial showed an unclear difference of 1.96 % ± 4.8 between N-3 PUFA and placebo. This measure was the most variable of the protocol as the participants were fatigued from previous tests. This was an inherent challenge of our protocol, as we wanted to measure the effect of N-3 s on all tests requiring all energy systems from power (ATP) to aerobic endurance. Nevertheless, 50 % of participants on N-3 PUFA improved time trial performance while only 33 % on placebo improved. Future evaluation of N-3 PUFA supplementation should focus on one or two of these areas to tease out the effects, as the combination clearly increases variability of results.

### Mechanism of action

Our data indicates N-3 PUFA supplementation improved peripheral neuromuscular function with an unclear effect on central neuromuscular function. Animal model research has shown N-3 PUFAs can increase acetylcholine concentration and acetylcholinesterase activity at the neuromuscular junction [7]. This could increase the speed of action potential transmission across the neuromuscular junction, thereby increasing muscle activation and EMG.

N-3 PUFA supplementation is suggested to alter cellular membrane composition and fluidity [7]. This may enhance nerve conduction through lower nerve resistance or improved ion channel function from the regulation of mitogen activated kinase transcription factors [23]. In this study, N-3 PUFA supplementation may have altered muscle membrane dynamics. This could have enhanced muscle action potential conduction through the working muscle. Altered membrane dynamics could have mitigated muscle damage resulting from the 10RM squat test. A reduction in damage might explain the attenuated Wingate test performance observed in the N-3 PUFA group. Similarly, reduced muscle damage could maintain muscle action potential conduction, thereby maintaining muscle excitation contraction coupling and ultimately muscle force generating capacity [24].

### Supplement duration

This study used a short-term supplementation period (21-days), as findings could be applied to support athlete adaptations during training camps or a pre-competition taper. Other studies with ergogenic application have demonstrated positive results in terms of reducing post-exercise inflammation [8], increasing muscle protein synthesis [25] or adaptations to training [6] using supplement periods of 4–12 weeks. While our supplementation period was shorter than other studies, it has been shown that 7 days is sufficient to increase plasma EPA concentrations [26] and attenuate muscle soreness from eccentric exercise [27]. Furthermore, 14 and 21-days of supplementation in humans [28] and pigs [9], was sufficient to increase muscle N-3 PUFA concentration.

Our supplementation protocol used a realistic N-3 PUFA supplement dose of 1115 mg^.^d^−1^ essential fatty acids, compared to previous N-3 PUFA intervention studies using arguably super-physiologic doses of 3000 – 8000 mg^.^d^−1^ [9, 25, 29, 28]. Therefore, we feel these results have a high level of applicability to athletes and the training population, as high doses of N-3 PUFA may not be tolerable by individuals over the long term and also can be expensive.

This is one of the first exercise studies to use a seal oil N-3 PUFA supplement in contrast to the more commonly used fish oil N-3 PUFA. Seal oil N-3 PUFA supplementation is the sn-1/3 position of N-3 fats compared to the sn-2 of fish oil. This difference has been shown to promote more rapid digestion of seal oil N-3 leading to subsequent higher incorporation into non-hepatic tissues [30]. Participants in this study ingested supplements in oil form to allow for potential sublingual absorption of the sn-3 N-3 [14]. This method of uptake may have increased the total bioavailable N-3 PUFAs as transit through the stomach can increase oxidation [8] and ultimately enable greater uptake into muscle and nervous tissue.

### Strengths and limitations

Several limitations became apparent throughout this study. Using participants from an endurance training background but different sports, yielded differences in athletic abilities. While triathletes excelled at the endurance portions of the experiment (time trial), they had more variability in the 10 RM squat test. It would be advisable that future studies of a similar nature recruit athletes from the same, or similar, athletic backgrounds and focus on a specific energy system.

## Conclusion

In conclusion, 21-days of N-3 PUFA supplementation increased plasma EPA N-3 PUFA concentration. Neuromuscular function was improved through increased muscle activation and sprint cycling performance was maintained from attenuated Wingate percent power drop. Our data provide a basis for further investigation of the effects of N-3 PUFA supplementation on the neuromuscular system and as an ergogenic aid for trained individuals.

## Abbreviations

DHA: Docosahexaenoic acid
DPA: Docosapentaenoic acid
EMD: Electromechanical delay
EMG: Electromyography
EPA: Eicosapentaenoic acid
MVC: Maximum voluntary contraction
N-3 PUFA: Omega-3 polyunsaturated fatty acid
PLA: Placebo
RFD: Rate of force development
RM: Repetition maximum
TT: Time trial
VL: Vastus lateralis
